# Supplementary material for: The Construction and Exploration of a Comprehensive MicroRNA Centered Regulatory Network in Foxtail Millet (Setaria italica L.)
Source: Front Plant Sci. 2022 May 6;13:848474. doi: 10.3389/fpls.2022.848474 (PMC9121102; doi:10.3389/fpls.2022.848474)
Supplement: Supplementary file 6 [file Data_Sheet_6.zip › Sit-miRNovel5_Seita.5G319100.1_3319_TPlot.pdf]

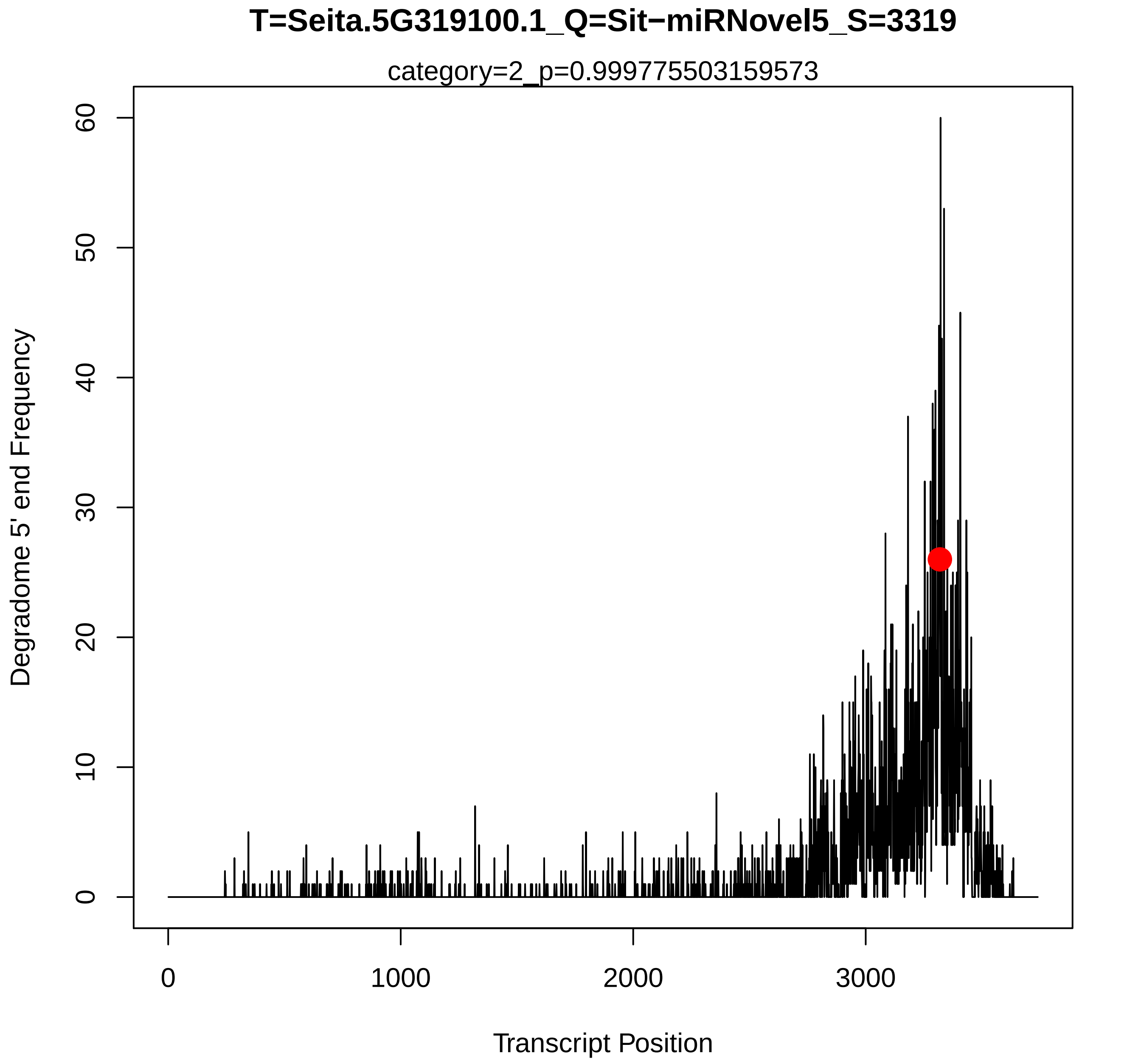
T=Seita.5G319100.1_Q=Sit−miRNovel5_S=3319
category=2_p=0.999775503159573
Degradome 5' end Frequency
0
10
20
30
40
50
60
0
1000
2000
3000
Transcript Position
